# Absence of Scaffold Protein Tks4 Disrupts Several Signaling Pathways in Colon Cancer Cells

**DOI:** 10.3390/ijms24021310

**Published:** 2023-01-09

**Authors:** Mevan Jacksi, Eva Schad, László Buday, Agnes Tantos

**Affiliations:** 1 Institute of Enzymology, Research Centre for Natural Sciences, 1117 Budapest, Hungary; 2 Doctoral School of Biology, Institute of Biology, ELTE Eötvös Loránd University, 1117 Budapest, Hungary; 3 Department of Molecular Biology, Semmelweis University Medical School, 1094 Budapest, Hungary

**Keywords:** Tks4, signal transduction, long non-coding RNA, gene regulation, EGFR signaling, epithelial–mesenchymal transition, Frank–ter Haar syndrome

## Abstract

Tks4 is a large scaffold protein in the EGFR signal transduction pathway that is involved in several cellular processes, such as cellular motility, reactive oxygen species-dependent processes, and embryonic development. It is also implicated in a rare developmental disorder, Frank–ter Haar syndrome. Loss of Tks4 resulted in the induction of an EMT-like process, with increased motility and overexpression of EMT markers in colorectal carcinoma cells. In this work, we explored the broader effects of deletion of Tks4 on the gene expression pattern of HCT116 colorectal carcinoma cells by transcriptome sequencing of wild-type and Tks4 knockout (KO) cells. We identified several protein coding genes with altered mRNA levels in the Tks4 KO cell line, as well as a set of long non-coding RNAs, and confirmed these changes with quantitative PCR on a selected set of genes. Our results show a significant perturbation of gene expression upon the deletion of Tks4, suggesting the involvement of different signal transduction pathways over the well-known EGFR signaling.

## 1. Introduction

Signaling cascades, orchestrated by the sequential phosphorylation events of the participants, are master regulators of cell behavior, controlling, among others, proliferation, migration, developmental programs, differentiation, and apoptosis. The key enzymes that govern these signaling pathways are protein kinases, which play essential regulatory roles in cells. Inadequate functioning of these enzymes is involved in the development of various diseases, including different cancer types [1]. However, important as kinases are, they are not sufficient in themselves to maintain an intact signaling cascade. Scaffold proteins that are capable of interacting with and bridging diverse pathway components are equally indispensable for correct signaling to occur [2].

One such scaffolding protein is tyrosine kinase substrate 4 (Tks4), a member of the scaffold protein family that is encoded by the SH3PXD2B gene (gene ID: 285590). Tks4 is a PX domain-containing protein with four Src homology 3 (SH3) domains [3] and is one of the five members of the p47 organizer protein family that modulates intracellular signaling [4] through the epidermal growth factor receptor (EGFR). In response to epidermal growth factor (EGF) stimulation, Tks4, which is mainly localized in the cytoplasm in quiescent cells, migrates to the membrane [3], where the PX domain is responsible for membrane binding [5], while the SH3 domains mediate interactions with various protein partners [6], with the protein kinase SRC among them. SRC is activated by binding to the intracellular region of EGFR [7] and the activated SRC phosphorylatesd Tks4. Phosphorylated Tks4 in turn can anchor activated SRC for an extended time, enabling the phosphorylation of downstream targets [8].

The importance of this scaffolding is underlined by the fact that the absence of Tks4 is responsible for the development of a severe genetic disease called Frank–ter Haar syndrome [9,10,11,12]. Tks4 deficiency also causes aberrant development in mice, resulting in phenotypic changes similar to those seen in Frank–ter Haar patients [13,14].

EGFR signaling is involved in diverse cellular processes, including proliferation and motility, and it is also implicated in the pathogenesis of various human cancers [15]. EGFR activation induces cell proliferation through the Ras/Raf/MAPK signaling cascade [16], increases migration and invasion via the PI3K/AKT pathway [17], and regulates calcium-mediated signaling through phospholipase Cγ [18] and cell survival by the Nck/PAK cascade [19]. As Tks4 serves as a platform for interacting pathway components in the cell membrane, it has the potential to influence all of the signaling events connected to EGFR. Consequently, Tks4 has been implicated in a wide array of cellular and developmental processes, including cell migration, podosome formation, mesenchymal stem cell differentiation, adipose tissue beiging, and bone trabecular formation [3,9,14,20,21,22].

Multiple pieces of evidence indicate the involvement of Tks4 in the pathogenesis of different cancers. As a result of its role in podosome formation, it has been suggested to increase metastatic capability of oral squamous cell carcinoma [23], hepatocellular carcinoma (HCC) [24], melanoma [22], and lung cancer [25]. Tks4 also appears to be involved in the reactive oxygen species generation and invadopodia formation in colon cancer [26]. These observations commonly denote decreased metastatic activity and reduced proliferation of the cancer cells in the absence of Tks4. A previous study on colorectal carcinoma showed a somewhat surprising result of Tks4 knockout, resulting in increased motility and an epithelial-mesenchymal transition-like behavior [27]. It was suggested that the permanent absence of Tks4 in epithelial cells activates an EMT-like program.

In order to extend our understanding of the effect of Tks4 deletion in colorectal cancer epithelial cells, we attempted to provide a comprehensive list of genes that are affected by the deletion of Tks4 and link them to specific signal transduction pathways. The results help us draw a clearer picture of the involvement of Tks4 in several signaling cascades and take us closer to understanding its intricate functions in malignant processes.

## 2. Results

### 2.1. Changes in Cell Motility and Proliferation

**Figure 1:**
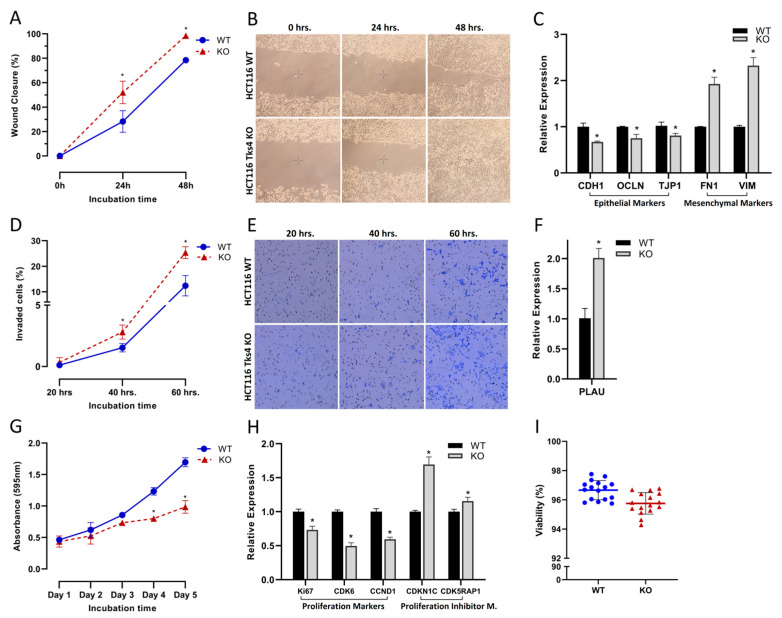
Effect of Tks4 on the motility, invasion, and proliferation. (**A**) Migration speed of the wild-type (blue) and Tks4 KO (red) HCT116 cells. (**B**) Wound healing assay showing cell migration of wild-type HCT116 cells (upper row) and Tks4 KO HCT116 cells (lower row). (**C**) Expression levels of epithelial and mesenchymal marker proteins. (**D**) Invasion capacities of wild-type (blue) and Tks4 KO (red) HCT116 cells. (**E**) Invasion assay of the wild-type (upper row) and the Tks4 KO (lower row) HCT cells. (**F**) Overexpression of the invasion marker PLAU (urokinase-type plasminogen activator), based on Q-PCR results. (**G**) Proliferation of the wild-type (blue) and Tks4 KO (red) HCT116 cells. (**H**) Changes in expression of proliferation markers and inhibitors upon the deletion of Tks4 based on the Q-PCR experiments. (**I**). Viability of the wild-type (blue) and the Tks4 KO (red) HCT116 cells. Results significant at *p* > 0.005 are marked with an asterisk.

The loss of Tks4 induced various morphological changes in the HCT116 cells, in line with the previously observed EMT-like behavior [27]. The migration speed of the cells was significantly higher in the absence of Tks4 (Figure 1A,B). In a wound healing assay, the wound closure was 20% higher after 24 and 48 h in the Tks4 KO cells, compared to the wild-type. Quantitative measurement of the EMT markers revealed that the expression level of most of the epithelial markers (CDH1, OCLN, and TJP1) decreased, while the mesenchymal markers (FN1 and VIM) [28] presented an elevated expression (Figure 1C).

In addition to the migration speed, the invasion capacity was also increased (Figure 1D,E) when Tks4 was absent. Similar to the migration assay, a 25% increase was observed in the percentage of the invaded cells in the Tks4 KO cell line (Figure 1D), while the expression level of PLAU (urokinase-type plasminogen activator), an invasion marker [29], increased more than two-fold (Figure 1F), with other invasion markers were also overexpressed in Tks4 KO cells (Supplementary Figure S1A and Table S1).

Interestingly, this increase in cell motility and invasion capacity was accompanied by a significant reduction in the cell proliferation rate of the cells lacking Tks4 (Figure 1G). In a 5-day long proliferation assay, WT and KO cells showed a similar rate of proliferation up to the second day after adhesion, which dramatically slowed down after the third day, reaching a 42% reduction on the fifth day. Gene expression analysis revealed that all the major activators of cell proliferation were downregulated in the Tks4 KO cells, while simultaneously the inhibitors were upregulated (Supplementary Figure S1B and Supplementary Table S1). The expression levels of Ki-67 [30] and CCND1 [31] proteins were reduced by about 25% and 40%, respectively, while CDK6 [32] decreased by 50%. As opposed to these, CDKN1C (p57Kip2) [33] and CDK5RAP1 [34] expressions increased by 70% and 15%, respectively (Figure 1H). While the viability of the Tks4 KO cells was slightly reduced compared to the WT cells (Figure 1I and Supplementary Figure S1C), the reduction was less than 1%. Although statistically significant, this small change cannot be responsible for the almost 50% reduction in proliferation rates.

### 2.2. Changes in mRNA Levels

**Figure 2:**
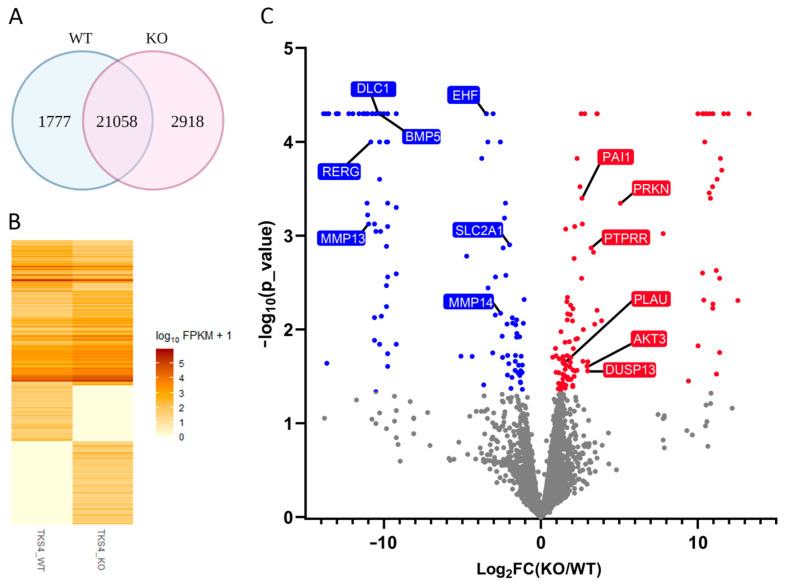
Changes in gene expression in Tks4 KO cells. (**A**) Venn diagram of the annotated genes expressed in WT and Tks4 KO cells. (**B**) Heat map of the mRNA expression levels in the Tks4 KO and the wild-type HCT116 cells. Each band corresponds to a specific gene. Darker colors represent higher expression of the specific gene. (**C**) Volcano plot of the differentially expressed mRNAs in the Tks4 KO cells. Each dot represents a specific gene. Gray: non-significant changes, red: upregulation, blue: downregulation.

In order to gain a deeper insight into the broadscale changes in gene expression patterns induced by the removal of Tks4, we carried out RNA sequencing of total RNA samples (Supplementary Tables S2 and S3). Out of the 25753 annotated genes (coding and non-coding together), the majority was shared between the two cell lines, about two thousand and almost three thousand were expressed only in the wild-type or the KO cells, respectively (Figure 2A). We identified 14327 protein coding genes that showed differential expression in the Tks4 KO cell line, which indicates large-scale reorganization of the gene expression regulation upon the loss of Tks4 (Figure 2B).

Significantly upregulated genes include PLAU (urokinase-type plasminogen activator) and its inhibitor, PAI1 (plasminogen activator inhibitor 1), AKT3, PRKN, and DUSP13 (Figure 2C). All of these genes have been implicated in cancer progression and metastasis, their overexpression generally resulting in more aggressive phenotypes [35,36,37,38] and apoptosis [39].

Significantly downregulated genes in Tks4 KO cells include EHF, RERG, MMP13, MMP14, BMP5, and DLC1 (Figure 2C). These genes are involved in epithelial differentiation and proliferation [40] and tumor development, with low expression connected to poor prognosis [41,42,43].

**Figure 3:**
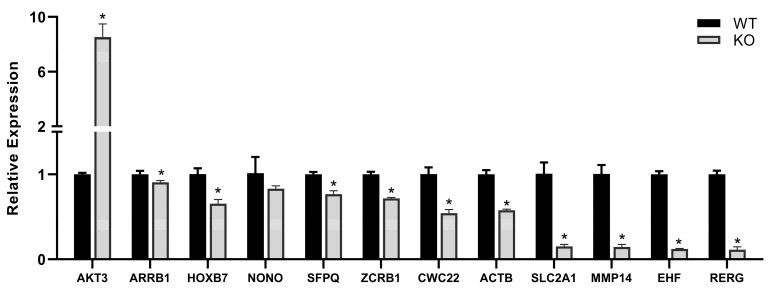
Differences in the expression levels of mRNAs, based on RT-qPCR results. Results significant at *p* > 0.005 are marked with an asterisk.

Quantitative PCR (RT-qPCR) results confirmed the gene expression changes detected by NGS and even extended the findings (Figure 3). Genes with relevant functions that showed marked expression changes in the Tks4 KO cells, but were not categorized as significant by the algorithm, were selected in addition to the significantly affected genes. Remarkably, AKT3 (RAC-gamma serine/threonine-protein kinase) expression increased seven-fold in the Tks4 KO cells (the fold change in FPKM value was 6.7, see Supplementary Table S2). The glucose transporter SLC2A1 and other members of the protein family (Figure 2C and Figure 3 and Supplementary Table S2) were strongly downregulated in the Tks4 KO cells, implying that Tks4 is important for the signaling pathways that maintain cancer progression.

Beta-actin (ACTB) is also downregulated upon the deletion of Tks4 as shown by RT-qPCR (Figure 3) and Western blotting (Supplementary Figure S2).

Genes involved in mRNA splicing and maturation (NONO, SFPQ, CWC22, and ZCRB1) [44,45,46,47] are downregulated too (Figure 3), suggesting that not only gene expression regulation, but also mRNA processing, is disturbed in the absence of Tks4. If we consider the wholescale changes in expression of proteins involved in mRNA splicing, we can see that almost all of them show lower expression in the Tks4 KO cells (Supplementary Figure S3 and Supplementary Table S1).

**Figure 4:**
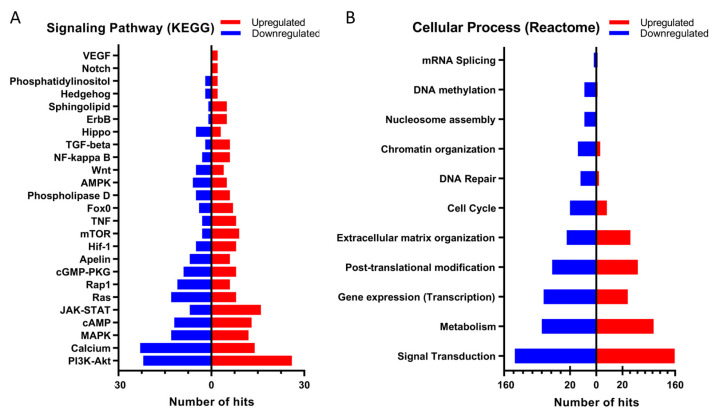
The effect of Tks4 deletion on the signaling pathways (**A**) and cellular processes (**B**). Only genes that showed at least two-fold change in expression (either down- or up-regulation) were considered in the analysis. Upregulation is marked in red, while downregulation is marked in blue.

KEGG signaling pathway analysis (Figure 4A) shows that the PI3K/AKT signaling pathway is the one that is most affected by the deletion of Tks4, as expected from the known function and molecular interactions of Tks4 [4]. A large-scale destabilization of this cascade is indicated by the almost equal number of down- and up-regulated genes, which is also the case for the MAPK and the cAMP signaling networks. Genes related to the JAK/STAT pathway are mostly upregulated, while genes involved in the calcium and Ras signaling are mostly downregulated. Although they are not among the most affected pathways, mTOR, TNF, TGF-β, and NF-kappa B networks appear to be upregulated in the cells that express no Tks4. VEGF, Notch, and Hedgehog pathways.

It has to be emphasized that downregulation or upregulation of repressor genes can have opposite effects on the functioning of the whole pathway, meaning that the final outcome of the dysregulated gene expression cannot be assessed only by the number of repressed or overexpressed genes.

In line with the extensive changes in gene expression, several cellular processes are affected in the Tks4 KO cells (Figure 4B). Not surprisingly, signal transduction had the most genes with at least a two-fold up- or down-regulation. Cellular metabolism, transcription, and posttranslational modification processes were also greatly affected. While several genes participating in extracellular matrix organization were downregulated, the upregulated genes proved to be more significant. Interestingly, DNA repair and chromatin organization also suffered downregulation, showing an impaired capacity of the Tks4 KO cells to react to cellular stress.

Tks4 seems not to be heavily involved in the formation and maintenance of cancer stem cells, as stemness markers (CD133, CD144, CD24, CD166, CD44, CD29, ALDH1, LGR5, PCGF1, and CXCR4) [48] are not significantly affected by the deletion of Tks4 (Supplementary Table S2), many of them are even absent from our dataset.

### 2.3. Changes in lncRNA Levels

During the past few decades, it has been established that not only protein coding genes play important roles in the regulation of cellular processes, but non-coding RNAs (ncRNAs) are just as relevant. In order to better understand the perturbations that Tks4 knockout induces, we analyzed the expression of long non-coding RNAs (lncRNAs) in both WT and Tks4 KO cells. LncRNAs are RNA molecules longer than 200 nucleotides that do not carry protein coding capacity [49]. Several lncRNAs have been shown to be crucial players in different cancers [50,51,52,53] and many of them are considered as new biomarkers [54].

**Figure 5:**
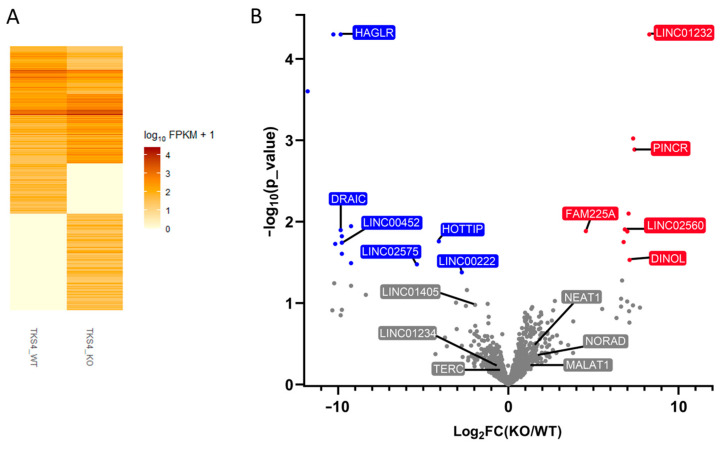
Changes in lncRNA expression in Tks4 KO cells. (**A**) Heat map of the lncRNA expression levels in the Tks4 KO and the wild-type HCT116 cells. Each band corresponds to a specific gene. Darker colors represent higher expression levels. (**B**) Volcano plot of the differentially expressed lncRNAs in Tks4 KO cells. Each dot represents a specific gene. Gray: non-significant changes, red: upregulation, blue: downregulation.

Our analysis identified 1893 differentially expressed lncRNAs in the Tks4 KO cells compared to the wild-type (Figure 5A, Supplementary Table S3). As the functional annotation of lncRNAs is still in its developing state, many of the identified lncRNAs do not have clear function. Nevertheless, we found several lncRNAs already implicated in cancer development to be overexpressed or downregulated upon the deletion of Tks4.

LncRNAs regulated by p53 and induced by DNA damage (PINCR, DINOL, and NEAT1) [55,56,57] appear to be overexpressed in the Tks4 knockout cells, together with the EMT-related LINC02560 [58] and LINC01232, which are implicated in cancer progression [59,60].

Tks4 deletion resulted in the downregulation of several lncRNAs as well, including LINC02575, HAGLR, LINC00222, and HOTTIP, involved in the KRAS [61], MAPK/AKT [62], Wnt [63], and c-Myc [64] pathways, respectively.

**Figure 6:**
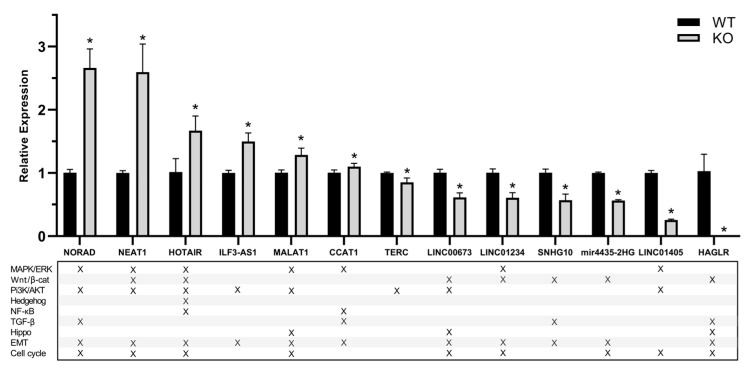
Changes in the expression of selected lncRNAs measured by RT-qPCR. The lower panel shows the signaling pathway and the cellular process they are known to be involved in. Results significant at *p* > 0.005 are marked with an asterisk.

LncRNAs present in high copy numbers proved to be non-significantly different between the wild-type and Tks4 KO cells based on the FPKM values, even though their expression levels were strongly affected (Figure 5B). RT q-PCR experiments on the other hand provided significant differences between the presence of most of the lncRNAs with EMT and cancer-related functions (Figure 6). NORAD and NEAT1 showed the strongest overexpression in the Tks4 KO cells, with a 2.5-fold increase in expression. For comparison, the FPKMs for the two lncRNAs showed 1.3 and 1.6-fold changes, respectively (Supplementary Table S3). TERC, LINC01234, and LINC01405 on the other hand were downregulated (Figure 6) (fold changes based on FPKM values were 0.79, 0.8, and 0.25, respectively, Supplementary Table S3).

LncRNAs tend to have multiple cellular partners and act through a variety of different molecular mechanisms. Therefore, they are intimately involved in several signal transduction pathways (lower panel of Figure 6) and their altered expression can cause severe perturbations in the biological functions controlled by them.

The changes in expression levels based on the RT q-PCR results indicate that lncRNAs involved in the PI3/Akt, MAPK, and WNT/β-catenin pathway are mostly affected in the Tks4 KO cells. These lncRNAs are also heavily involved in EMT and cell cycle regulation and their altered expressions correlate with a profound imbalance in these processes in the absence of Tks4.

## 3. Discussion

The above detailed results indicated that Tks4 deletion prompts excessive changes in the gene expression landscape of HCT116 cells, many of which are directly involved in signal transduction pathways. Since the most important function of Tks4 is to serve as a scaffold for the signaling pathways downstream of EGFR, this is an expected outcome. However, our results show that the effects of Tks4 KO reach far further than the destabilization of this cascade.

In the following, we discuss the findings related to the most important features of the Tks4 KO cell line.

### 3.1. The PI3K/AKT Pathway

**Figure 7:**
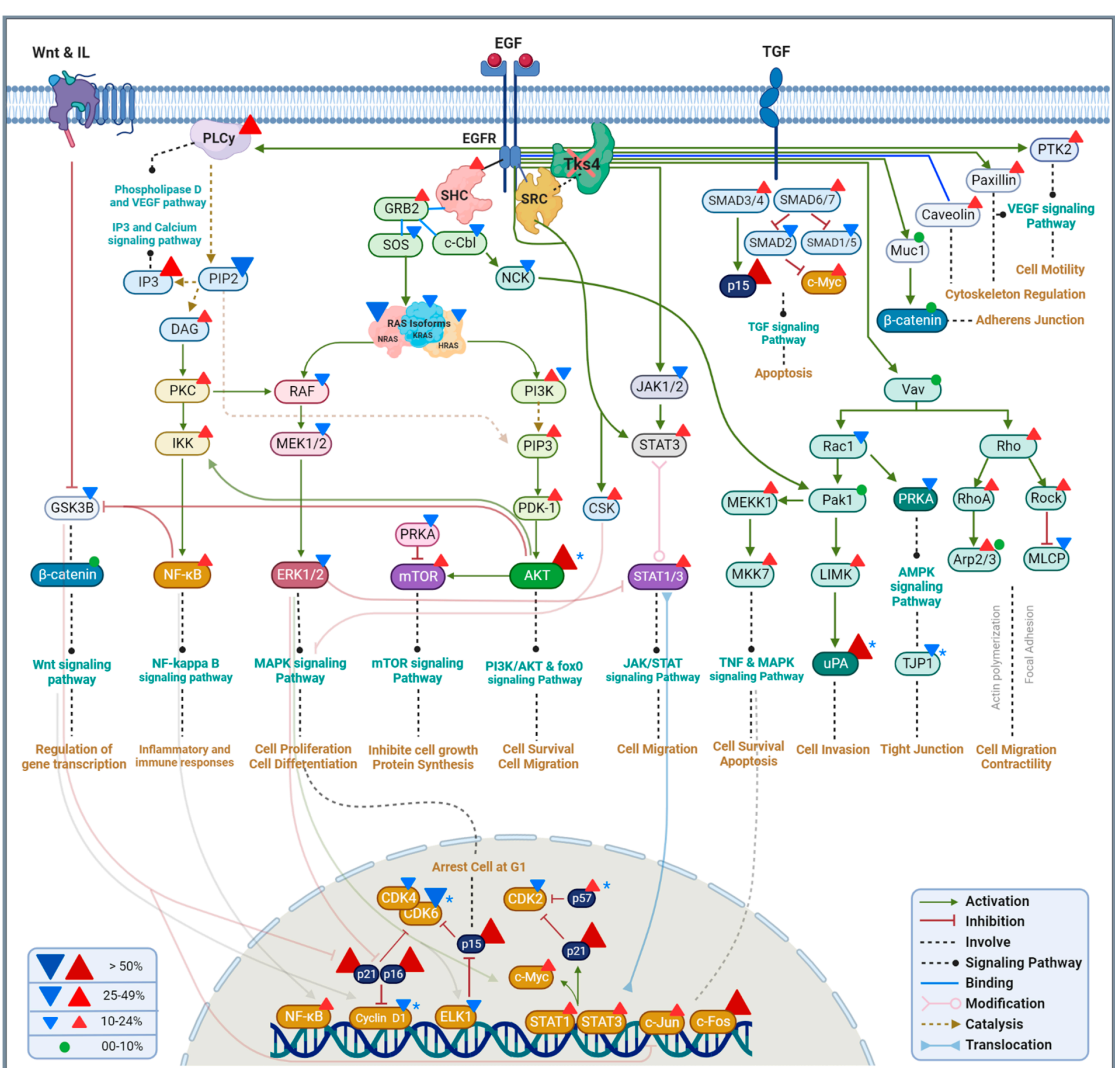
Schematic representation of the signaling pathways affected by the deletion of Tks4. Expression changes in the genes are represented by triangles (red for upregulation and blue for downregulation. When both are present, they refer to the expression changes in different subunits). Gene expression changes that were confirmed by RT-qPCR are marked with blue asterisks. Dark colors represent central genes in a specific pathway.

We observed an overexpression of several genes related to the PI3K/Akt pathway, including the significantly upregulated PLAU, PAI1, and AKT3 genes (Figure 2 and Figure 7 and Supplementary Table S2). This pathway is well-known to be overactivated in cancers [65] and its upregulation in the Tks4 KO cells can explain the observed increase in their migration capacity. In terms of specific genes, PAI1 specifically is known to increase tumor cell migration and invasion [36], while simultaneously decreasing cell proliferation [66], which was detected in the Tks4 deleted cells (Figure 1).

The elevated expression of AKT3 also indicates an increased activity of this pathway; furthermore, high AKT3 expression was associated with increased EMT of colorectal cancer cells [67], suggesting that loss of Tks4 may accelerate progression of this cancer type. Previous observations also suggest that AKT3 negatively controls the expression of MMP13 [37], which is in line with our observations here (Figure 2C), with AKT3 significantly overexpressed and MMP13 consequently downregulated.

In addition to the protein coding genes, several lncRNAs implicated in the regulation of the PI3K/AKT pathway have also shown altered expressions in the Tks4 KO cells. HOTAIR [68], NORAD [69], NEAT1 [70], and MALAT1 [71,72] are all overexpressed, probably further increasing the activity of the signaling cascade.

### 3.2. The MAPK/ERK Pathway

The MAPK/ERK signaling pathway is mainly connected to cell proliferation, differentiation, development, inflammatory responses, and apoptosis. It is one of the most affected pathways upon the deletion of Tks4. Its key regulatory proteins, such as RAS, RAF, MEK1/2, and ERK1/2 were mostly downregulated in the Tks4 KO cells (Figure 7 and Supplementary Table S2) which manifests as a reduction in the proliferation of these cells. EHF (ETS homologous factor), a transcription factor in the MAPK signaling pathway that is proposed to play a determining role in epithelial differentiation and proliferation [40], was also downregulated in the Tks4 KO cells on the mRNA (90% reduction) and on the protein level (35% reduction) (Figure 2 and Figure 3 and Supplementary Figure S2) providing an explanation for the epithelial cell line-specific changes observed earlier [27]. The difference in mRNA and protein abundances may be a result of other regulatory mechanisms, such as the efficiency of translation, which may serve as a buffer compensating for the loss of mRNA.

RERG (Ras-related and estrogen-regulated growth inhibitor) also showed a reduced expression in the Tks4 KO cells. It has been found that RERG is hypermethylated and downregulated in colorectal carcinoma [73,74], and the fact that loss of Tks4 resulted in a further reduction in RERG expression is in line with the increased invasion capacity of the Tks4 KO cells.

Among the non-coding RNAs, HAGLR (or HOXD-AS1) lncRNA was also downregulated upon the deletion of Tks4 (Figure 5B and Figure 6). The downregulation of this RNA facilitates the migration of colorectal carcinoma cells through a HAGLR-HOXD3-integrin β3 regulatory axis [62]. HAGLR appears to suppress HOXD3 transcription, and in its absence, HOXD3 can induce the MAPK pathway by increasing integrin β3 transcription. Other studies have argued for an oncogenic role of HAGLR, as it can act as a competing endogenous RNA for miR-217 [75], promoting tumor progression in CRC. In our Tks4 deleted cells, the lower expression of HOXD-AS1 was accompanied by an increase in both HOXD3 and integrin β3 expression (Supplementary Table S2), suggesting that the HAGLR-HOXD3-integrin axis is connected to Tks4.

The general suppression of this pathway has also been indicated by the lower expression of the KRAS-regulated lncRNA LINC02575 (or KIMAT1) [61] in the KO cell line.

In contrast to the downregulated genes, some lncRNAs involved in the MAPK/ERK pathway seem to be overexpressed in the Tks4 KO cells. These include HOTAIR [76], NORAD [77], NEAT1 [78,79], CCAT1 [80], and MALAT1 [81].

### 3.3. Epithelial Mesenchymal Transition

One of the most interesting observations regarding the effects of the Tks4 knockout is the induction of an incomplete EMT-like phenomenon [27] and the increase in the invasion capacity of the Tks4 KO cells, because the well-known role of Tks4 in podosome formation would suggest the opposite outcome. Nevertheless, the changes in gene expressions provide a molecular foundation for these events.

Many of the canonical EMT markers show varied expression levels in the Tks4 KO cells. Proteins characteristic to epithelial cell types such as E-cadherin, laminin, MUC-1, and claudins are mostly unaffected on the RNA level (Supplementary Table S2), while others, such as nectin 3 and tight junction proteins, are downregulated. Some alterations to this can occur on protein levels, as it was shown that E-cadherin is internalized and degraded in the Tks4 cells [27]. In parallel, mesenchymal markers, such as vimentin, Zeb1/2, and Snail2 are upregulated based on the NGS data, but not all of them, as Twist1 and N-cadherin are downregulated. These changes indicate an incomplete EMT-like process and correspond to the results published earlier with the same Tks4 KO cell line [27].

However, our transcriptome analysis provided further details on the changes in the expression levels of coding and non-coding genes related to EMT.

Matrix metalloproteinases (MMPs) are heavily involved in the induction of EMT [82] and mostly appear to be overexpressed in the Tks4 KO cells, while MMP13 and MMP14 are downregulated (Figure 2C and Figure 3 and Supplementary Table S2).

BMP5 (bone morphogenetic protein 5) downregulation has been implicated in the EMT processes in colon adenocarcinoma [42]. Its significant downregulation in Tks4 KO cells underlines the pro-EMT phenotype induced by the deletion of Tks4. It also indicates a dysregulation of the BMP/Smad signaling pathway, as BMP5 was suggested to act via this cascade [83]. Interestingly, BMP5 was also found to be involved in the regulation of adipogenesis in Syrian hamsters [84], a process that is affected in the Tks4 KO mouse model [14].

Apart from the protein coding genes, several lncRNAs have been shown to be involved in the process of EMT.

Many pro-EMT lncRNAs were overexpressed in the Tks4 KO cells (Figure 6 and Supplementary Table S3). Examples include NORAD [85,86,87], NEAT1 [88], CCAT1 [89], LINC02560 [58], and HOTAIR [90]; however, similar to the coding genes, others, such as LINC00673 [91], HOTTIP [92], and MIR4435-2HG [93] were downregulated.

The expression of SNHG10, which was shown to suppress EMT in epithelial ovarian cancer [94], was reduced in the Tks4 KO cells, probably resulting in a pro-EMT effect.

### 3.4. Podosome Formation

Importantly, many of the established invadopodia and podosome formation markers (MMP14, Zyxin, [23], Arp2/3, and N-WASP [95]) were downregulated in the Tks4 KO cells, in accordance with the protein playing an important role in podosome formation. Nevertheless, several others such as Lasp1 [96], fascin, dynamin, vinculin, paxillin, and paladin [97] were upregulated in various levels. Among the lncRNAs, invadopodia-related examples AFAP1-AS1 [98] and SH3PXD2A-AS1 [99] were also downregulated, with the targets of SH3PXD2A-AS1, p57, and KLF2 simultaneously upregulated. MALAT1 [100] and LINC00511 [101] on the other hand were upregulated. Taken together, these results show that the deletion of Tks4 results in a general downregulation of genes involved in podosome and invadopodia formation, while the exceptions probably indicate that there are other, Tks4-independent mechanisms regulating these processes.

### 3.5. Proliferation, Senescence and Apoptosis

Somewhat surprisingly, the changes in the migration and invasion properties of the Tks4 KO cells were accompanied by a significant reduction in the proliferation rate. These processes are generally considered to be parallelly increased in EMT [102]. Nevertheless, not only the morphological observations, but also the gene expression changes confirmed the downregulation of Cdks and the upregulation of cyclin inhibitor proteins such as p15, p16, p21, and p57 (Figure 7 and Supplementary Table S2), which causes cell cycle arrest. Other participants of the MAPK cascade, such as different RAS isoforms, RAF, MEK1/2, and ERK1/2 were similarly downregulated in the Tks4 deficient cells. The upregulation of specific components of the PI3K/AKT signaling pathway, such as PIP3, PDK-1, and AKT, results in increased cell migration and survival.

**Figure 8:**
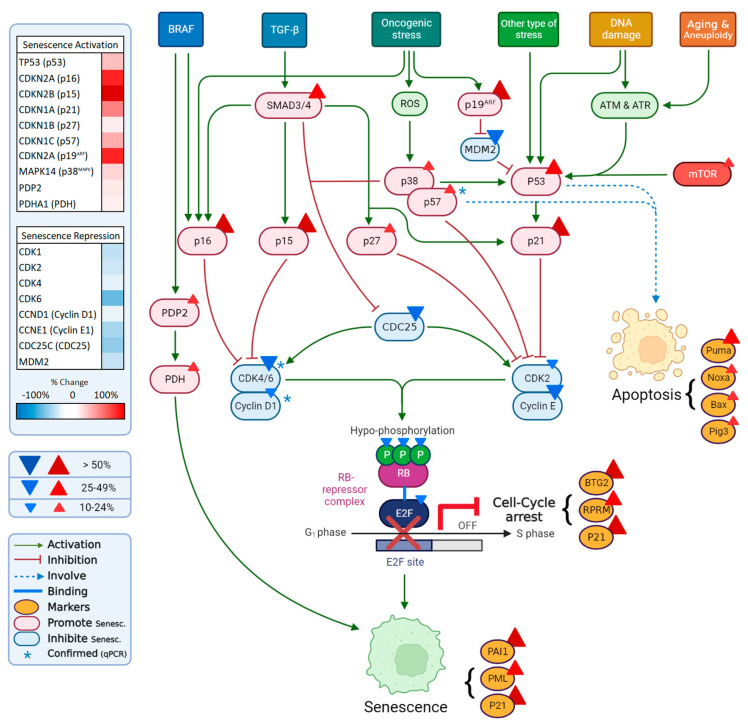
Gene expression changes related to cellular senescence and apoptosis. Expression changes in related genes based on the NGS data are shown on the left upper panel. Changes that were confirmed by RT-qPCR are marked with blue asterisks.

In addition to the reduction in proliferation rate, the apoptosis and senescence pathways were apparently also activated upon the deletion of Tks4 (Figure 8 and Supplementary Table S1).

The expression of p53, the central protein safeguarding cellular integrity [103,104], was upregulated, while Mdm2 [105], its regulatory protein, was downregulated. This, in turn, activated apoptotic pathways, represented by the overexpression of apoptosis markers PUMA (BBC3), Noxa, Bax, and Pig3 (Supplementary Table S2). P53 overexpression was also mirrored in the upregulation of p53-dependent lncRNAs, such as PINCR, DINOL, and NEAT1. MALAT1 may also participate in the apoptotic processes, as it was overexpressed in the Tks4 KO cells and is capable of inducing apoptosis [106].

Changes in lncRNAs linked to apoptosis also indicate the activation of the p53 pathway. DNA damage related lncRNAs, induced by p53-like PINCR [55], DINOL [56], and NEAT1 [57] were all upregulated upon the deletion of Tks4 (Figure 5B and Figure 6 and Supplementary Table S3).

DINOL is capable of binding and stabilizing p53 and PINCR is responsible for the control of the expression of 11 genes involved in cell cycle arrest and apoptosis [55]. In our sequencing data, most of these genes (especially SNAI2, BTG2, and EPAH2) showed a moderate upregulation in the Tks4 KO cell line (Supplementary Table S2), supporting a similar regulatory connection and a link between Tks4 and the p53 pathway.

This is an apparent contradiction to previous observations of EMT being an efficient method for cells to escape apoptosis [107,108]. A possible explanation of our result is offered by the observation that in CRC cells, EMT is capable of inducing apoptosis through the downregulation of KLF5 and the subsequent switch of Sox4 from antiapoptotic to proapototic roles [109]. While our experiments were not aimed at the detailed study of Sox4 function in Tks4 KO cells, KLF5 expression was repressed in Tks4 deficient cells, indicating an analogous regulation.

In parallel with the increased apoptosis, key senescence markers were also upregulated in the Tks4 KO cells (Figure 8). Serpine 1 is an important factor responsible for the regulation of replicative and cellular senescence [110], and it was highly upregulated in the Tks4 KO cells, as well as PML [111] and p21 [112]. Interestingly, senescence-related lncRNAs, such as NORAD [113,114], MALAT1 [115], SNHG12 [116], and SALRNA1 [117], were upregulated in the Tks4 KO cells, and their overexpression was generally connected to the inhibition of senescence. Since these lncRNAs participate in several other cellular processes through their multiple molecular partners, it is possible that their main functions in the Tks4 KO cells are manifested through other pathways, not related to senescence.

The results summarized above clearly demonstrate that loss of Tks4 has a profound effect on the EGFR signaling. As a result of the central role that EGFR plays in the initiation of other signaling cascades, such as the RAS/RAF/MEK, PI3K/AKT, PLC, and JAK/STAT pathways [118], this results in global gene expression changes and leads to marked alteration in the behavior of cells. When interpreting the results, it must be taken into consideration that protein levels may show alterations to mRNA abundances, just as in the case of EHF, which may somewhat attenuate the effect of the gene expression changes. On the other hand, this is not true for the non-coding RNAs that are implicated in these pathways, which also show severely altered expression patterns in the Tks4 deleted cells.

The determination of the causal role of the observed gene expression changes and the exact downstream effectors that mediate these alterations will require further experimental work. Nevertheless, our observations provide a detailed picture of the molecular background of the physiological effects attributed to Tks4 [4] and deepen our understanding of the role Tks4 plays in the crosstalk of different signaling pathways.

## 4. Materials and Methods

### 4.1. Cell Culture

The Tks4 KO HCT116 cell line was generated as described in a previous publication in 2019 [27]. The absence of Tks4 was confirmed in the protein level by Western blotting and immunohistochemistry (Supplementary Figure S4A,B).

McCoy’s 5A medium (Life Technologies, Paisley, UK) supplemented with 10% fetal bovine serum (FBS, Life Technologies, Paisley, UK) and 1% penicillin/streptomycin (Life Technologies, Paisley, UK) was used to cultivate HCT116 cells. All cell cultures were maintained in a humidified environment containing 5% CO_2_ at 37 °C. Cell number and viability were determined with the TC20 Automated Cell Counter (Bio-Rad, Hercules, CA, USA). The morphology was analyzed using an Axiovert 25 inverted microscope (Carl Zeiss Microscopy, Jena, Germany).

### 4.2. Cell Proliferation Assay

For the cell proliferation assay, 1 × 10^5^ cells were seeded into a 24-well plate containing 1 mL of McCoy’s 5A complete medium in each well. Following adhesion, cells were washed twice with 1X PBS, fixed with 10% formalin solution, and marked as day 0. From days 2 to 5, cells were fixed daily. Cells were washed with PBS and then left to dry at room temperature. An amount of 500 μL of 0.5% crystal violet (C0775, Merck KGaA, Darmstadt, Germany) was used to stain the cells. After washing, crystal violet was dissolved by adding 10% acetic acid (Merck KGaA, Darmstadt, Germany) to each well. The remaining cell mass was determined as the total absorbance measured at 595 nm using a SYNERGY MX plate reader (BioTek Instruments, Windooski, VT, USA).

### 4.3. Cell Migration Assay

Cell migration assays were performed using a culture-insert 2 well in µ-Dish (Ibidi GMBH, Gräfelfing, Germany). According to the manufacturer’s protocol, 7 × 10^4^ cells were seeded in culture-insert dishes and incubated until they reached confluency. Then, the plastic inserts were removed and the dishes were washed with PBS and re-equipped with 1 mL McCoy’s 5A complete medium (Life Technologies, Paisley, UK) before being incubated at 37 °C to allow for wound closure. Images were taken by a LEICA DMi1 inverted microscope (Leica Microsystems GmbH, Wetzlar, Germany) at 0 h, 24 h, and 48 h.

### 4.4. Transwell Invasion Assay

Cell invasion assays were performed using BioCoat^®^ Matrigel^®^ Invasion Chambers (354480, Corning Inc., Corning, NY, USA). Cells were seeded in the upper chambers at a density of 2.5 × 10^4^ in McCoy’s 5A serum-free medium and incubated for 48 h. A 5% FBS solution was added to the lower chamber as a chemoattractant. The cells on the upper surface of the inserts were removed by a cotton-tipped swab after 20, 40, and 60 h of incubation time, and the cells on the bottom surface were fixed with 100% methanol and stained with crystal violet. Images were taken with a LEICA DMi1 inverted microscope. The invasion efficiency was determined by counting and analyzing the number of invading cells in 10 random fields at 10× objective lens magnification.

### 4.5. Viability Assay

The viability of HCT116 WT and Tks4 KO cells was determined by the addition of equal volumes of 0.4% trypan blue dye to the cells suspended in serum-free complete McCoy’s 5A medium, and the mixtures were incubated for up to three minutes at room temperature. Cells were counted by a hemocytometer counter using a LEICA DMi1 inverted microscope. The ratio of viable cells was calculated by dividing the number of viable cells by the number of total cells.

### 4.6. Total RNA Extraction and Real-Time Quantitative PCR

TRIzol™ Reagent (Life Technologies, Carlsbad, CA, USA) was used to lyse HCT116 cell lines. A Direct-zol^®^ MiniPrep kit (Zymo Research, Irvine, CA, USA) was used to isolate the total RNA. DNAse I (Zymo Research, Irvine, CA, USA) treatment for 15 min removed DNA contamination. For reverse transcription, a First Strand cDNA Synthesis Kit for RT-PCR (AMV) (Roche, Basel, Switzerland) was used to generate complementary DNA (cDNA) from 1 µg of total RNA. With the QuantStudio™ 5 and 6 pro Real-Time PCR System (Life Technologies, Paisley, UK), the real-time PCR experiments were carried out by using TaqMan™ Fast Advanced Master Mix (Life Technologies, Paisley, UK). Three replicates were used to determine the relative expression based on the 2^−ΔΔCt^ method to determine fold changes. Applied Biosystems provided the probes for TaqMan^®^ assays, which are listed in Supplementary Table S4.

### 4.7. RNA Sequencing

The total RNA of HCT116 cells was isolated using a Direct-zol^®^ MiniPrep kit after cell lysis with TRIzol™ Reagent was performed. DNA contamination was removed after 15 min of treatment with DNAse I. The quality and quantity of the RNAs were determined using a Nanophotometer IMPLEN (iBiotech, Prague, Czeck Republic). After rRNA depletion, 1 µg of RNA of each sample was used for whole transcriptome sequencing on an Illumina NovaSeq 6000 high throughput NGS platform (Novogene, Cambridge, UK).

### 4.8. RNA Sequencing Data Analysis

We prepared two biological replicates for the WT and three replicates for the KO samples. The obtained 150 bp paired-end reads were aligned to the GRCh38 human transcriptome using HISAT2 (2.2.1 release) [119]. Aligned reads were manipulated (converted to BAM format, sorted, and indexed) with samtools (1.12 release) [120]. Transcriptome assembly and differential expression analysis were performed using the Cufflinks program package (cufflinks, cuffmerge, cuffquant, and cuffdiff) (2.2.1 release) [121]. Changes in expression levels were calculated based on the differences in FPKM (fragments per kilobase of exon per million mapped fragments) numbers of the WT and KO samples. Differences were given as base two logarithms of the ratio between the KO and the WT samples (log_2_Fold change).

Volcano plots were created by using GraphPad Prism 8.0.2 (GraphPad Software, San Diego, CA, USA) software and heatmaps were created with the CummeRbund R package (R version 4.2.1, cummeRbund 2.38.0) [122].

### 4.9. Protein Extraction and Western Blot Analysis

Proteins were extracted using RIPA buffer (150 mM NaCl, 1% NP-40, 1% sodium deoxycholate, 0.1% SDS, 25 mM Tris-HCl, pH 7.6) with addition of a protease inhibitor cocktail (10 μL/mL) (A32965, Life Technologies, Paisley, UK) after washing with cold PBS. Cell debris was pelleted by centrifugation at 18,000× *g* for 10 min at 4 °C. A Pierce™ BCA Protein Assay Kit (23227, Life Technologies, Paisley, UK) was used to determine the protein concentrations of the supernatants. An amount of 25 µg of the protein sample was separated using a 4–20% gradient TGX gel (4561094, BioRad, Hercules, CA, USA) and then transferred to a nitrocellulose membrane (BioRad, Hercules, CA, USA). After blocking for one hour at room temperature with tris-buffered saline containing 0.05% Tween-20 (TBS-T) with 5% non-fat milk, the membrane was rinsed three times with TBS-T and incubated overnight at 4°C with selected primary antibodies: Tks4 (A303-437A, Merck KGaA, Darmstadt, Germany), EHF (PA5-84038, Life Technologies, Paisley, UK), ACTB (MA1-140, Life Technologies), and GAPDH (PA1-16777, Life Technologies). Goat anti-rabbit IgG (H+L) superclonal secondary antibody (A27036, Life Technologies) diluted in TBS-T at a concentration of 1:20,000 and goat anti-mouse IgG (H+L) secondary antibody (31431, Life Technologies) diluted in TBS-T at a concentration of 1:10,000 were used for the detection of the specific bands. Membranes were developed with enhanced chemiluminescence (ECL) reagent (1705061, BioRad, Hercules, CA, USA) and the generated signals were captured using the ChemiDocTM imaging system (BioRad, Hercules, CA, USA). Densitometry of the Western blot bands was carried out with ImageJ version 1.53 (Bethesda, MD, USA) software [123].

### 4.10. Immunocytochemistry and Fluorescence Microscopy

Cells were seeded at a density of 35,000 cells per well onto a rounded cover slip (VWR, Leicestershire, UK). At 80% confluence, the medium was decanted and the cells were washed three times with 500 µL of pre-warmed PBS. The cells were fixed in 4% para-formaldehyde solution for 15 min at room temperature, washed again, and then blocked for one hour at room temperature using 500 µL of complete blocking solution (0.2% gelatin, 2% BSA, 5% FBS, and 0.1% TritonX-100 completed with 1X PBS). Afterwards, they were incubated overnight at 4 °C with the appropriate primary antibodies as listed in the subsection detailing Western blotting. Following the incubation, the cells were rinsed with PBS three times and then incubated for one hour at room temperature with the rabbit IgG (H+L) cross-adsorbed secondary antibody Alexa Fluor 488 (A-11008, Life Technologies, Paisley, UK). Nuclei were stained with DAPI (Merck KGaA, Darmstadt, Germany). Images were captured using a ZEISS LSM-710 confocal microscopy system (Carl Zeiss microscopy Gmbh, Jena, Germany). ZEN 3.2 software was used to process the images (Carl Zeiss microscopy Gmbh, Jena, Germany).

### 4.11. Statistical Analyses

Statistical significance was determined by using a multiple unpaired Student’s *t*-test in GraphPad Prism version 8.0.2 (GraphPad Software, San Diego, CA, USA).

## Data Availability

The datasets generated and analyzed during the current study are available from the corresponding author on request.

## Supplementary Materials

The following supporting information can be downloaded at: https://www.mdpi.com/article/10.3390/ijms24021310/s1.
