# Supplementary material for: Absence of Scaffold Protein Tks4 Disrupts Several Signaling Pathways in Colon Cancer Cells
Source: Int J Mol Sci. 2023 Jan 9;24(2):1310. doi: 10.3390/ijms24021310 (PMC9861885; doi:10.3390/ijms24021310)
Supplement: Supplementary file 1 [file ijms-24-01310-s001.zip › Supplementary Figures_Revised.pdf]

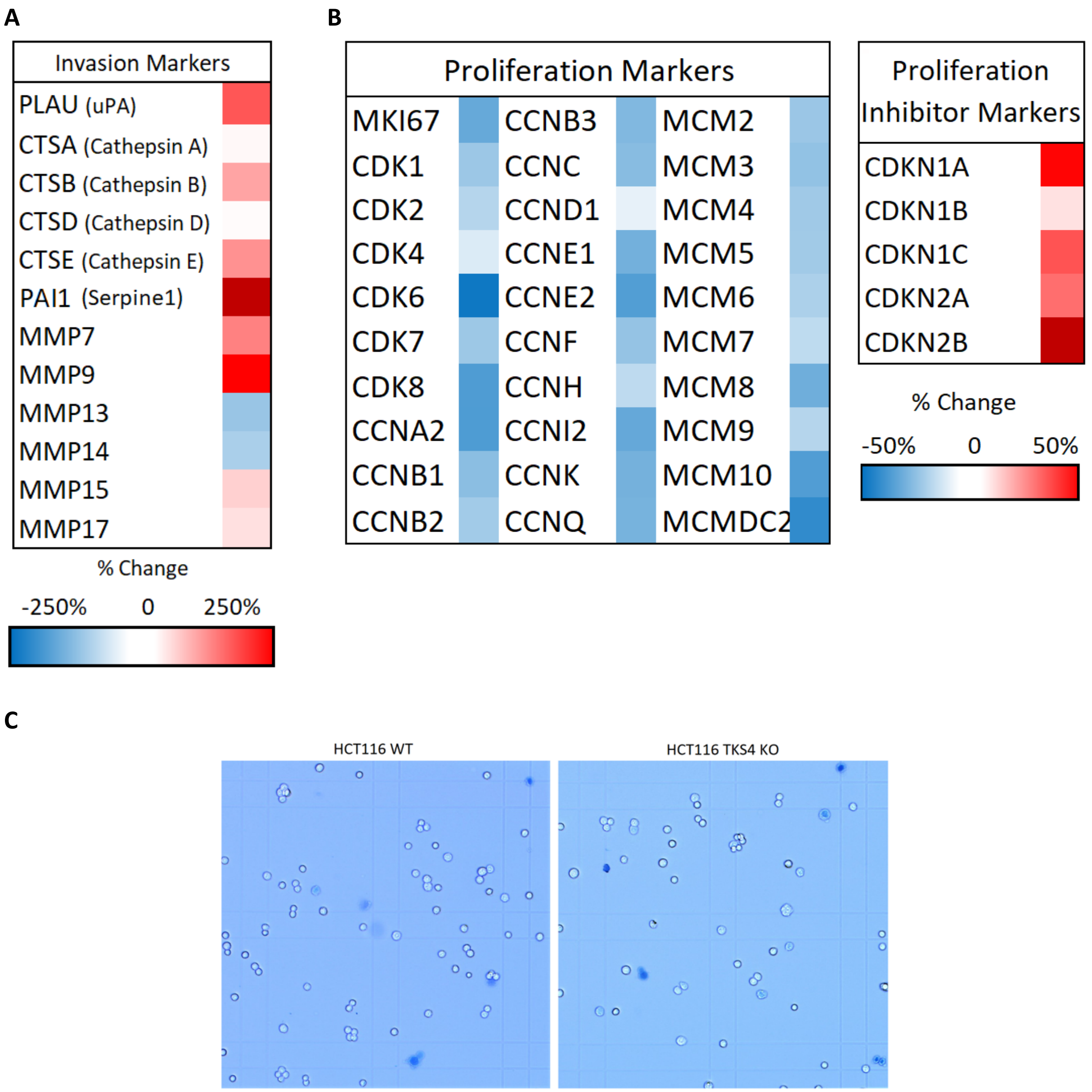

Figure S1. Proliferation and invasion markers, and viability of the Tks4 KO cells A. Changes in the expression levels of invasion markers based on the NGS data. Red: overexpression; blue: downregulation. Darker colors represent higher differences. **B.** Changes in the expression levels of proliferation markers based on the NGS data. Red: overexpression; blue: downregulation. Darker colors represent higher differences. **C.** Viability Assay of HCT116 WT and Tks4 KO cells under the light microscope stained with Trypan Blue. Viable cells are clear, dead cells are blue.

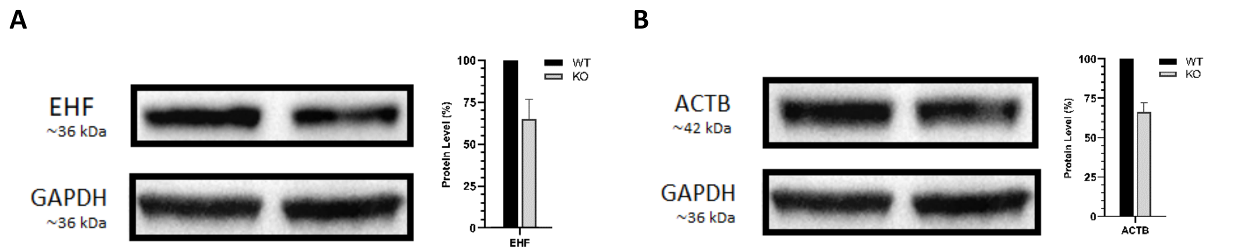

Figure S2. Protein abundances in WT and Tks4 KO HCT116 cells. A. Abundance of EHF protein in the wild type (left lane) and in the Tks4 KO cells (right lane). The bar graph represents the intensity differences between the bands, normalized to GAPDH expression. **B.** Abundance of ACTB protein in the wild type (left lane) and in the Tks4 KO cells (right lane). The bar graph represents the intensity differences between the bands, normalized to GAPDH expression.

Splicing Factors				Connected to Splicing			
Gene name	KO/ WT	Gene name	KO/ WT	Gene name	KO/ WT	Gene name	KO/ WT
SF1		SFSWAP		HNRNPA1		HNRNPH3	
SF3A1		SRSF1		HNRNPA1L2		HNRNPK	
SF3A3		SRSF3		HNRNPA2B1		HNRNPL	
SF3B1		SRSF4		HNRNPA3		HNRNPLL	
SF3B2		SRSF6		HNRNPAB		HNRNPM	
SF3B3		SRSF7		HNRNPC		HNRNPR	
SF3B4		SRSF8		HNRNPD		HNRNPU	
SF3B5		SRSF9		HNRNPDL		FUS	
SF3B6		SRSF10		HNRNPF		CCAR1	
SFPQ		SRSF11		HNRNPH1		WBP4	

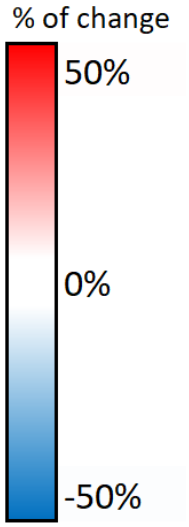

Figure S3. Changes in the expression levels of mRNA splicing markers based on the NGS data. Red: overexpression; blue: downregulation. Darker colors represent higher differences.

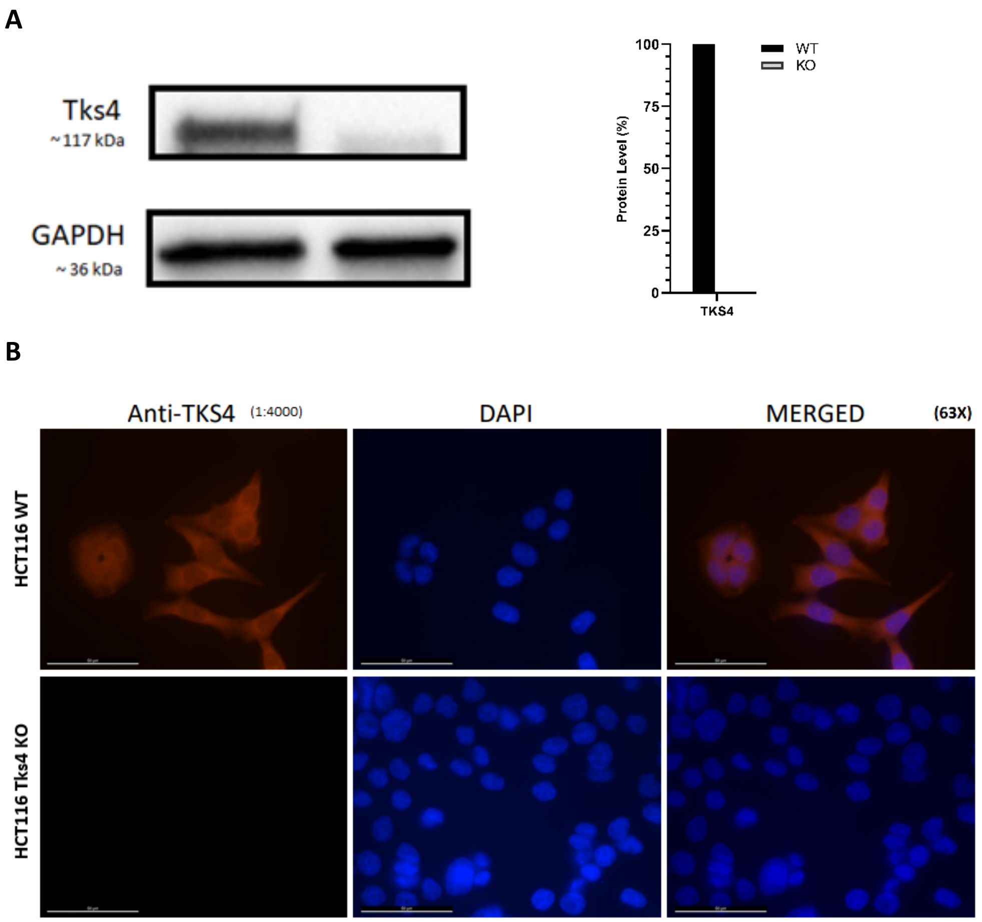

Figure S4. Tks4 abundances in the WT and KO HCT116 cells. A. Abundance of Tks4 protein in the wild type (left lane) and in the Tks4 KO HCT116 cells (right lane). The bar graph represents the intensity differences between the bands, normalized to GAPDH expression.**B.** Representative images of wild type (upper row) and Tks4 KO HCT116 cells stained with anti-Tks4 antibody.
